# The Treatment of Post-Partum Hæmorrhage

**Published:** 1901-04-27

**Authors:** 


### THE TREATMENT OF POST-PARTUM HÆMORRHAGE.

To all general practitioners the treatment of post-partum hæmorrhage is a matter of constant interest, for, however fortunate a man may have been, he never knows that his very next patient may not be attacked with hæmorrhage of that terrible and uncontrollable severity which fortunately the careful practitioner but seldom sees. It has been too much the habit of writers on the subject to recommend a series of measures to be tried in succession. Influenced, no doubt, by the fact that in the majority of cases the hæmorrhage, bad as it may be for the moment, does in the end stop—although whether owing to the treatment adopted may sometimes be questioned—various more or less simple proceedings are recommended to be tried in the first place, and if they fail we are told to pass on to more elaborate methods, all of which is hardly logical. In some cases indeed it is disastrous, for whenever the more simple methods fail time, that most valuable asset, is wasted, and the measures ultimately resorted to act at a disadvantage.

At a discussion on the treatment of post-partum hæmorrhage which recently took place at the Manchester Clinical Society Mr. Stanmore Bishop, in introducing the subject, laid great stress upon the fact that the methods in common use, and generally recommended by the authorities, depend for their efficacy either on producing contraction of the uterus or on setting up coagulation in the bleeding vessels. He laid great emphasis upon this, and quoted authorities to show that if the means generally recommended failed to set up coagulation, and failed to produce contraction, they failed altogether, leaving the patient with open vessels to bleed to death.

In regard to the efforts made to produce coagulation, and especially in regard to the injection of perchloride of iron, he asks why obstetricians ignore the teachings of general surgery, and have recourse to styptics in their efforts to stop bleeding from large vessels; a course the futility of which has been demonstrated again and again by surgical experience. We come back then to this, that the one sole means on which we depend is the production of contraction in the uterine muscle. Whether we have recourse to flicking the abdomen with wet towels, to forcible compression of the uterus between the hands, one within and the other without, or to compression by both hands outside, or between packing within and the hands outside, or to the use of ergot or strychnine, in all case alike unless the uterus can be thereby persuaded to contract failure must result. Why then does the uterus sometimes refuse to contract, do what we will, and stimulate it as we like? The answer is that it is tired, and that until it has had rest it *cannot* contract; and unfortunately rest is just what we cannot give it while the patient is bleeding to death. Is there no means then by which we can, just for a few minutes, give this uterus rest, and can hold the bleeding in check while that rest is obtained? This is the question which Mr. Stanmore Bishop asked his auditors, and this question he answered by saying that we must simply apply in this situation the rules accepted in regard to hæmorrhage in other situations. What, he says, do we do when confronted with hæmorrhage from any other part of the body? Surely to control the bleeding from the veins we raise the part, and to control that from the arteries we hold the vessel. Applying this to obstetrics the great thing is to take the readiest and quickest means of placing the patient in the Trendelenburg position, one way of doing which is to run a table under the bed, allowing the foot of the bedstead to rest upon it. By this means one may at once place the patient at an angle of 45° or 50° with the ground. But a chair with footstools or books piled upon it will do. Having then controlled venous bleeding, the next point is the arteries, and if one remembers that all the arteries from which the blood is coming arise from one source, namely the aorta, one is led directly to the idea of compressing the aorta. Five-sixths of the blood going to the uterus reach that organ through the uterine arteries, which arise from the lower part of the aorta, and so are capable of being controlled by pressure upon any portion of its trunk, while so far as the blood reaching the uterus through the ovarian arteries is concerned that is unimportant as a cause of hæmorrhage, and is likely to be of use in feeding the uterine muscle and enabling it to recuperate.

What Mr. Stanmore Bishop recommends then is that the closed fist should be applied with its ulnar surface resting upon the aorta as it lies over the left side of the vertebral column, and that just sufficient pressure should be exerted obliquely backwards and towards the right to enable it to compress the vessel against the unyielding surface beneath. At any point accessible over the course of the abdominal aorta this compression can be exerted, and there is always, he says, a sufficient extent available to enable one to vary the site of impact. The prolonged tension of the abdominal walls during the pregnancy has rendered them lax and unresisting, so that now that the uterus is empty the aorta is readily found and as readily controlled. Whilst this compression is kept up the uterus may be cleared of clot, portions of placenta, membranes, &c. As to the length of time for which it may be necessary to keep up the pressure, that must be governed by the same principles as would apply in general surgery. In general surgery the artery is controlled until the ligatures are made secure. In obstetrics the aorta must be held until the uterus secures the vessel by contracting. This in practice will be found to coincide pretty much with the restoration of firm pulsation in the aorta against the compressing hand. When it is found that the uterus is contracting as it ought to do then the pressure may be gradually removed. This then is what has to be done in presence of post-partum hæmorrhage: Control the venous flow by raising the foot of the bed, and control the arteries by pressure on the aorta until once again the uterine muscle is enabled by its contraction to prevent further loss of blood. So much for what Mr. Stanmore Bishop said, which, it may be confessed, carries with it a good deal of conviction, although most accoucheurs will probably call to mind certain adipose ladies in whom it must have been very difficult to find and compress the aorta.

In the discussion on this paper the older methods on which Mr. Stanmore Bishop had thrown discredit found supporters, and various objections were raised to the proposal to compress the aorta. It was to be noted, however, that those who objected to his proposal confirmed his statement that in trusting to other methods they put their faith in their power of inducing contraction of the uterus; and it is easy to see that if such contraction can be obtained there is an end of the difficulty. The whole question, indeed, seems to hinge upon this: Can the uterus always be made to contract? If it can, there are half-a-dozen methods of stimulating it so to do. But if it be true, as is asserted, not by Mr. Stanmore Bishop alone, that there are cases in which by no available amount of stimulation can the uterus be made to contract; and if it be true, as is stated by Mr. Stanmore Bishop, that it is easy to control bleeding, even under such circumstances, by pressure upon the aorta, then it does become a serious question whether, on the occurrence of post-partum hæmorrhage, one should not at once adopt the plan of pressing upon the aorta, believing, as we all do, that if the uterus can but be given time it will by its own contraction do all that is required. This is the matter that wants bringing to the test of a large experience in many hands.

